# Effects of Long-Continued Use of Chloral

**Published:** 1880-03

**Authors:** 


### ARTICLE VIII.

### *Effects of the Long-continued Use of Chloral.*

We learn from the *Lancet*, of January 17th, 1880, that at the general meeting of the Clinical Society of London, held on the ninth of that month, Dr. Farquharson read the Report of the Committee appointed to ascertain what deleterious effects follow the prolonged and continued use of chloral in ordinary doses. It stated that seventy special replies and three printed papers had been received in reply to nearly 1000 circulars distributed throughout the profession, followed a few months later by a second appeal, made public through the freely accorded medium of the medical press. Twenty-nine answers state that, after extensive experience of chloral in long continued doses, no ill effects have been observed. Ten of these correspondents enjoy the special opportunities for observation afforded by asylum practice, and Mr. Curgenven, Dr. C. T. Williams, Dr. W. Squire, Dr. Buzzard, Dr. Clifford Allbutt, and others, furnish cases in which chloral had been regularly and beneficially taken for periods varying from two to ten years. Before proceeding to analyze the replies received from those who had observed inconvenient effects to follow the use of chloral, the committee drew up a brief summary of what has already been recorded on the subject. Their special information has been arranged under the various headings of the schedule, thus: *A. Nervous System.* Fourteen answers record cases in which nervous debility, mental enfeeblement, and convulsive seizures appeared to follow the use of chloral, Dr. Maudsley, Dr. Clouston, and Dr. Lindsay expressing themselves as strongly opposed to its employment in insanity. *B. Circulatory System.* Two answers under this heading note some cardiac enfeeblement. *C. Digestive System.* Six replies mention digestive disturbance as occasionally following the administration of alcohol. *D. Cutaneous.* Nine correspondents give details of cases in which they observed itching of the skin, lichenous eruption, with deep flushing of face and head, following the taking of stimulants. *E.* Two replies indicate the possibility of urinary irritation being produced by chloral. Inquiry among some of the leading druggists of the metropolis has not established the probability that there is any remarkable abuse, by the public, of the facilities which they enjoy of purchasing for themselves any quantity of chloral. The drug, it may be mentioned, is not included by the legislature among those the sale of which is guarded by the name and address of the purchaser being required to be registered by the vendor. In conclusion, the committee expressed regret that, in spite of repeated appeals to individuals generally and to the profession, by the circular, and through the medical press, they have failed to obtain any more definite information than that contained in the preceding report; and, although the opinions expressed by numerous gentlemen of experience will, doubtless, be received with the respect which is their due, the committee would have been glad if more facts, from which definite conclusions might have been drawn, had been placed at their disposal.—*Med. and Surg. Report.*

---

### ARTICLE IX.

### *New York Odontological Society.*

